# Thermally Induced *lux*-Operon Promoter of *Photorhabdus hainanensis* and *Photorhabdus temperata*

**DOI:** 10.3390/microorganisms14092076

**Published:** 2026-09-17

**Authors:** Vadim V. Fomin, Valeriia O. Matveeva, Kamilla V. Mekhantseva, Daniil I. Sakharov, Sergei E. Spiridonov, Sergey V. Bazhenov, Nanette Hope Sumaya, Ilya V. Manukhov

**Affiliations:** 1 Moscow Center for Advanced Studies, Moscow 123592, Russia; 2 A.N. Severtsov Institute of Ecology and Evolution, Moscow 119071, Russia; 3 Department of Biological Sciences, Iligan Institute of Technology, College of Science and Mathematics, Mindanao State University, Iligan City 9200, Philippines

**Keywords:** *Photorhabdus*, luminescence, promoter, heat shock, RT-qPCR, *Escherichia coli* K12

## Abstract

The bioluminescence of entomopathogenic bacteria of the genus *Photorhabdus* was considered to be constitutive. However, it has recently been shown that under heat shock conditions, *lux*-operon is activated in *Photorhabdus temperata* cells. In the present study, bioluminescence thermoactivation was investigated in two isolated strains, *Photorhabdus hainanensis* FV2401 and *P. temperata* FV2402. The luminescence of the more thermotolerant *P. hainanensis* cells was activated at higher temperatures than that of *P. temperata*. At the maximum induction temperatures of 37 °C for FV2401 and 34 °C for FV2402, higher bioluminescence activation was observed in FV2401, whereas the increase in *lux*-genes mRNA levels was approximately the same in both strains. In *P. hainanensis*, elevated temperatures also affected the expression of the *hexA*, *madB*, and *csrB* genes, which are indirectly involved in luminescence in *Photorhabdus*. The heat-activated *lux*-operon promoter in *P. hainanensis* and *P. temperata* was determined. This is a σ^70^-dependent promoter that differs in its distance from the *luxC* start codon. When promoter variants with or without the 5′-UTR mRNA were transferred to the heterologous *Escherichia coli* system, heat activation of luminescence was retained in constructs containing only the promoter and was independent of σ^32^ and σ^E^, whereas constructs with the 5′-UTR exhibited repression of luminescence following thermoactivation. The obtained data on the temperature-induced promoter of the *lux*-operon can broaden understanding of the ecological and biological functions of *Photorhabdus* bioluminescence under heat shock conditions and provide a basis for the development of novel biosensors.

## 1. Introduction

*Photorhabdus* is a genus of terrestrial bioluminescent bacteria consisting primarily of symbiotic strains transmitted by juvenile *Heterorhabditis* nematodes. In 1999 [1], the genus was divided into three species: *Photorhabdus luminescens*, *Photorhabdus temperata*, and *Photorhabdus asymbiotica*. *P. luminescens* and *P. temperata* are symbionts of nematodes, whereas *P. asymbiotica* is a pathogenic species isolated from human wounds. Strains of *P. luminescens* isolated from warm and tropical regions remain viable at temperatures up to 35–39 °C, while strains of *P. temperata* isolated from temperate regions maintain viability up to 33–35 °C [1]. After many years of expanding the collection of isolated strains, the phylogenetic description of the genus *Photorhabdus* was revised in 2018 [2]. Therefore, currently, groups of species that are closely related to either *P. luminescens* or *P. temperata* in terms of maximum growth temperatures are distinguished.

The cells of symbiotic *Photorhabdus* species have two distinct phenotypic forms. In the non-luminous mutualistic M-form, bacterial cells reside in the intestines of nematodes prior to insect infection. When a host is infected, the cells switch to the pathogenic P-form, in which they intensively bioluminesce and produce a wide range of substances: insect toxins, immune inhibitors, antibiotics, pigments, and others [3,4,5]. It was previously demonstrated that the cell phenotypic form depends on the orientation of the *mad*-operon promoter: when the promoter is oriented toward the *mad*-operon, the *mad*-genes are transcribed, and cells exhibit M-form properties; when the orientation is reversed, the *mad*-genes are not transcribed, and cells are in the P-form [6,7].

Most bioluminescent bacterial species regulate *lux*-operon (*luxCDABE* genes) expression depending on cell density according to the principle of “quorum sensing” [8]. However, among bioluminescent bacteria, there are those that lack “quorum sensing” regulation, or in which the mechanisms of regulation are unknown, for example, the genera *Photorhabdus* and *Photobacterium* [8,9,10]. Nevertheless, induction of *lux*-gene transcription under heat shock conditions was previously shown in *P. temperata* FV2201 strain, which is viable at moderate temperatures, but the exact mechanism is unclear [11].

The only known regulator of *Photorhabdus* bioluminescence is the HexA master regulator. It is found in high concentrations in M-form cells and represses the expression of genes specific to P-form cells, including the *lux*-operon [6,12]. Thus, HexA functions as a regulator of the cell phenotypic state. However, the specific mechanism of bioluminescence control is unclear. HexA is believed to repress the *lux*-operon at a post-transcriptional level because changes at the transcription level were not observed [12]. In addition to *hexA*, *lux*-operon regulation by the CsrA-CsrB system has been proposed [13]. CsrA is an RNA-binding protein that typically suppresses the expression of target mRNA genes, whereas CsrB is a non-coding RNA that acts as an antagonist to CsrA [14]. Overexpression of *csrB*, but not *csrA*, increased *lux*-gene expression at the transcriptional level [13]. In *Escherichia coli*, induction of *csrB* transcription under heat shock conditions by a σ^E^-dependent mechanism has been shown [15].

Bacterial luciferase genes, and, sometimes, their native promoters or regulatory elements, are used to design biosensors that can detect small concentrations of specific substances and assess the toxicity of various compounds [16,17]. Studying thermal induction of the *Photorhabdus lux*-operon could help characterize new genetic elements for incorporation into novel biosensors that respond to heat shock through σ^32^- and σ^E^-independent mechanisms [11]. The role of bioluminescence in *Photorhabdus* bacteria is not clearly understood, but a recent study has demonstrated the effect of bioluminescence on multitrophic interactions [18]. When *Photorhabdus* bacteria and temperature as an abiotic factor are considered together, the focus is usually either on the effect of temperature on the performance of *Photorhabdus*-associated entomopathogenic nematodes as biocontrol agents [19,20,21], or on species closely related to *P. asymbiotica* in terms of their ability to act as human pathogens due to their viability at 37 °C [22,23,24]. Of particular interest is a study demonstrating activation of bioluminescence in *P. asymbiotica* during prolonged incubation at 37 °C compared to 28 °C, as well as a relatively short-term increase in bioluminescence in *P. luminescens* [22]. In that study, the authors suggested that increased oxygen consumption by luciferase reduces oxygen availability at 37 °C, thereby preventing microaerobic growth. It was also observed that bioluminescence is activated by the addition of human serum in the *P. luminescens* ZM1 strain [25].

The aim of this study was to investigate the mechanisms regulating *lux*-operon expression under heat shock conditions in two strains, *P. hainanensis* FV2401 and *P. temperata* FV2402, which were isolated from climatically distinct soil environments. To achieve the stated goal, luminescence induction of *Photorhabdus* cell suspensions and the transcriptional activity of the *luxC*, *luxA*, *luxE*, *hexA*, *madB*, and *csrB* genes at elevated temperatures were assessed, *lux*-operon promoter was identified, and its functional activity and the role of the 5′-UTR were characterized in *E. coli* cells.

## 2. Materials and Methods

### 2.1. Strains, Plasmids, and Oligonucleotides

Brief descriptions of the strains, plasmids, and oligonucleotides used in the study are given in Tables S1, S2, and S3, respectively.

The pLPhain, pSPhain, pFPhain, pFPtem, pOPhain, and pOPtem plasmids, based on pDEW201 [26], were constructed following the same scheme. All inserts were amplified from the corresponding total DNA templates of *P. hainanensis* FV2401 and *P. temperata* FV2402 using the following primer pairs, respectively: hainPLF and hainPLR; hainPSF and hainPSR; hainPLF and hainsPSR; pDPtemFor and pDPtemRev; hainPLF and hainPOR; pDPtemFor and temPOR. The pDEW201 plasmid was digested with *Eco*RI and *Kpn*I restriction enzymes. DNA fragments of the inserts and pDEW201 were purified by agarose gel extraction. The final plasmid constructs were assembled using the Gibson method [27]; then, *E. coli* MC1061 cells were transformed using CaCl_2_ [28].

### 2.2. Isolation of Nematodes and Photorhabdus-Associated Strains

Soil samples weighing 1–2 kg were collected along the Tabod River shoreline near Tipanoy Bridge in Santa Elena Municipality, Iligan City (Lanao del Norte Province, Philippines, coordinates: 8.195, 124.257) and transported to Mindanao State University-Iligan Institute of Technology (MSU-IIT). In the laboratory, the soil samples were distributed into 500 mL plastic containers. Ten caterpillars of the small wax moth *Achroia grisella* were added to the soil in each container. Containers were left in the laboratory at 25–28 °C for 3–4 days, during which the contents of the container and the condition of the caterpillars were examined daily. *A. grisella* caterpillars with a uniform reddish color were transferred to 40 mm diameter Petri dishes lined with filter paper. Tap water was added dropwise to the filter paper until the substrate was completely moistened, but without excess moisture. On days 6–7, Petri dishes with caterpillars were placed in a larger container with water at the bottom, preventing water from getting inside the dish with dead caterpillars. Between days 7 and 12, active migration of newly formed nematode larvae into the water at the bottom of a larger container was observed. The nematode suspension was poured into 175 mL culture flasks and stored at 14–15 °C. Sequencing of the ITS rDNA region (600 nucleotides, accession number PZ939114) of the nematodes confirmed the identification of the species as *Heterorhabditis indica*.

Soil samples were also collected in the Vladimir region of Russia (Pokrov district, near the Pernovo settlement) on a steep sandy slope above the Vol’ga River. The soil was distributed into 500 mL containers and transported to Moscow, where five caterpillars of the greater wax moth, *Galleria mellonella*, were added to each container. After 3–5 days of exposition at 22–25 °C, the contents of each container were examined for the presence of dead caterpillars with a reddish color. Further processing of nematodes was performed as described for Philippine nematodes. Sequencing of the ITS rDNA region (508 nucleotides, accession number PZ944513) of the nematodes confirmed the identification of the species as *Heterorhabditis megidis*.

*Photorhabdus* bacteria were obtained after reinfecting *G. mellonella* caterpillars with the isolated nematodes. The internal contents of the larvae, which had a reddish tint and exhibited noticeable luminescence in the dark, were plated onto solid LB medium. As a result, luminescent bacterial colonies of FV2401 and FV2402 were obtained, and their *16S* rRNA (650 nucleotides, accession numbers PZ938547 and PZ938552, respectively) and *infB* (832 nucleotides, accession numbers PZ944288 and PZ944289, respectively) genes were sequenced using primer pairs 16S_Ph_unidir, 16S_Ph_unirev and infBFor, infBRev, respectively (Table S3). The results of the phylogenetic analysis, which allowed determination of the species affiliation, are presented as trees in the Results Section and Figure S1.

### 2.3. Culture Conditions

*E. coli* and *Photorhabdus* bacteria were cultivated in LB medium (1% tryptone, 0.5% yeast extract, 1% NaCl) at 200 rpm at the temperatures indicated in the experiment. For solid medium in Petri dishes, LB was supplemented with up to 1.5% Bacto agar. *E. coli* MG1655 strains carrying the plasmids listed in Table S2 were grown in a medium supplemented with 100 μg mL^−1^ ampicillin.

The optical density of bacterial suspensions was measured using a KFK-2 photocolorimeter (ZOMZ, Sergiev Posad, Russia).

### 2.4. DNA Manipulation

Plasmid DNA extraction, bacterial transformation, 1% agarose gel electrophoresis, and DNA fragment extraction from the gel were performed according to the manual [28]. Restriction and ligation reactions were performed using Promega enzymes (Madison, WI, USA) according to the manufacturer’s protocols. Total DNA was extracted according to the method [29].

### 2.5. Luminescence Measurements

Luminescence measurements and data processing were carried out as described previously [11]. Cell suspensions were transferred into 1.5 mL microcentrifuge tubes, and then the tubes were placed in a Biotox-7BM luminometer (BioPhysTech, Moscow, Russia) to measure integral luminescence (in relative units) at room temperature (23 °C). Measurements were taken after at least 5 min at 23 °C to allow the samples to cool to standard conditions. Highly luminescent samples were diluted 10- or 100-fold to match the dynamic range of the luminometer.

Time-dependent kinetics of luminescence normalized to optical density were measured using a Synergy H4 plate reader (BioTek, Winooski, VT, USA) at either 37 °C or room temperature (23 °C), using three biological replicates with an initial OD_600_ ≈ 0.1.

### 2.6. Luminescence Data Processing

Luminescent bacterial cells were grown in liquid LB at 23 °C and 200 rpm to an optical density OD_600_ ≈ 0.1. Each cell suspension was then divided into equal portions, which were incubated for 3 h at the temperatures indicated in the experiments. After incubation, the luminescence and optical density of the cell cultures were measured.

To account for differences in growth rate at different temperatures, the luminescence values of a sample were normalized to the optical density as follows:

(1) $$NL=\frac{Lum}{{OD}_{600}}\times d$$

where NL—the normalized luminescence, Lum—the luminescence value, OD_600_—the optical density, and *d*—the dilution factor.

To compare samples at different temperatures, the induction coefficient was calculated as follows:

(2) $$K(T)=\frac{NL\left(T\right)}{NL(T_{0})}$$

where NL(T)—the normalized luminescence (1) of the sample at temperature T, K(T)—the luminescence induction coefficient at temperature T, and T_0_—the lowest temperature in the experiment (23 °C).

In experiments with ethanol, which stimulates heat shock, the final concentration of ethanol in the medium was varied instead of temperature, and the ethanol-free sample was used as the reference in the denominator of Equation (2). To minimize the effect of optical density on luminescence, bacterial cells were grown at 23 °C to an OD_600_ ≈ 0.1, and then divided into 200 μL aliquots in 1.5 mL microcentrifuge tubes. Ethanol was added, and incubation was continued at 23 °C without shaking. Under these conditions, the cell division rate was minimal; thus, the luminescence response was essentially independent of optical density and reflected the cellular response to ethanol toxicity. This response either increased, indicating activation of *lux*-operon expression, or decreased due to ethanol toxicity, as previously demonstrated with the pGrpE-lux biosensor [11]. Therefore, optical density was not taken into account in Equation (1) for the ethanol experiments.

### 2.7. RNA Extraction and RT-qPCR

*Photorhabdus* strains were grown at 23 °C and 200 rpm to an optical density OD_600_ ≈ 0.1. The cultures were then divided into equal portions that were incubated for 3 h at temperatures of 23 and 34 °C for FV2402, and 23 and 37 °C for FV2401, respectively. After incubation, the luminescence value and optical density of the suspensions were measured, and total RNA was extracted using the RNA Solo kit (Evrogen, Moscow, Russia) according to the manufacturer’s instructions. Total RNA concentration was assessed using a NanoPhotometer^®^ P 330 spectrophotometer (Implen, Munich, Germany).

RT-qPCR was performed using a Rotor-Gene Q amplifier (QIAGEN, Hilden, Germany) using the OneTube RT-PCR SYBR kit (Evrogen) according to the manufacturer’s instructions, but with the addition of reverse transcriptase at a final concentration of 0.25×. The target genes *luxC*, *luxA*, *luxE*, *hexA*, *madB*, and *csrB* were normalized to the housekeeping gene *16S*. To account for the possible effect of temperature on *16S* expression, all RT-qPCR reactions were performed using approximately 10 ng of total RNA as template, and the *C*_T_ values for the *16S* gene were compared between samples incubated at different temperatures. The observed variation in *16S* *C*_T_ values was minimal and more likely attributable to pipetting error than to temperature-dependent changes in *16S* expression. The corresponding primer pairs for amplification are listed in Table S3. Changes in RNA levels between samples at different temperatures are represented in calculated ΔΔ*C*_T_ values [30] as follows:

(3) $$\Delta \Delta C_{T}={\left(C_{T,Target}-C_{T,16S}\right)}_{Induced}-{\left(C_{T,Target}-C_{T,16S}\right)}_{Control}$$

where $C_{T,Target}$—the threshold cycle of the target gene, and $C_{T,16S}$—the threshold cycle of the *16S* gene. Biological samples incubated at 34 or 37 °C are indicated as “Induced”, and the corresponding samples incubated at 23 °C are indicated as “Control”.

The ΔΔ*C*_T_ values were calculated using the Rotor-Gene 6000 software 1.7 (Corbett Research, Sydney, Australia). Amplification mode: 50 °C for 15 min, 95 °C for 1 min, 45 cycles: 95 °C for 15 s; 57 °C for 20 s; 72 °C for 25 s. Melting curves: 50 °C for 1 min, from 50 to 94 °C in increments of 1 °C—5 s.

### 2.8. Lux-Operon TSS Identification

The *lux*-operon transcription start sites (TSS) of FV2401 and FV2402 were determined using the Step-Out RACE method [31] with the Mint RACE cDNA amplification set kit (Evrogen) and gene-specific primers (Table S3), according to the manufacturer’s instructions.

The following steps were performed sequentially. Total RNA was extracted from FV2401 and FV2402 after incubation at 23 °C or at 37 and 34 °C as described in RNA extraction section. First-strand cDNA was then synthesized from the extracted total RNA using the gene-specific primer RACE(1)luxC, Mint reverse transcriptase, and the PlugOligo-1 adapter (Evrogen). Next, double-stranded DNA was synthesized in 3 steps by nested PCR method using the first-strand cDNA as a template, with the sequential use of gene-specific primers RACE(2)luxC, luxC52R, and RACE(3)luxC paired with the corresponding end primers from the Mint RACE primer set kit (Evrogen). The resulting PCR products from the final step were purified by agarose gel extraction and sequenced using the RACE(3)luxC primer.

The TSS of the mRNA *lux*-operons in FV2401 and FV2402 were determined by alignment with upstream DNA sequences of the *luxC* using NCBI BLAST 2.17.0 [32] and separated from the primer nucleotides. The upstream DNA sequences of the *lux*-genes were determined by sequencing PCR products amplified from the FV2401 and FV2402 total DNA templates using primer pairs pluxFor, luxC52R and luxC52f, Ph_RluxC, respectively.

### 2.9. Statistics

All error bars represent the standard deviation calculated from at least three biological replicates. Statistical significance was in most cases assessed using a two-sample *t*-test, with the null hypothesis based on luminescence values at 23 °C or 0% ethanol, or by comparing values between two temperature groups or between strains. A one-sample *t*-test was used only to compare −ΔΔ*C*_T_ values against zero. Specific details of the statistical tests applied are provided in the Results Section.

## 3. Results

### 3.1. Bioluminescence Induction

**Figure 1:**
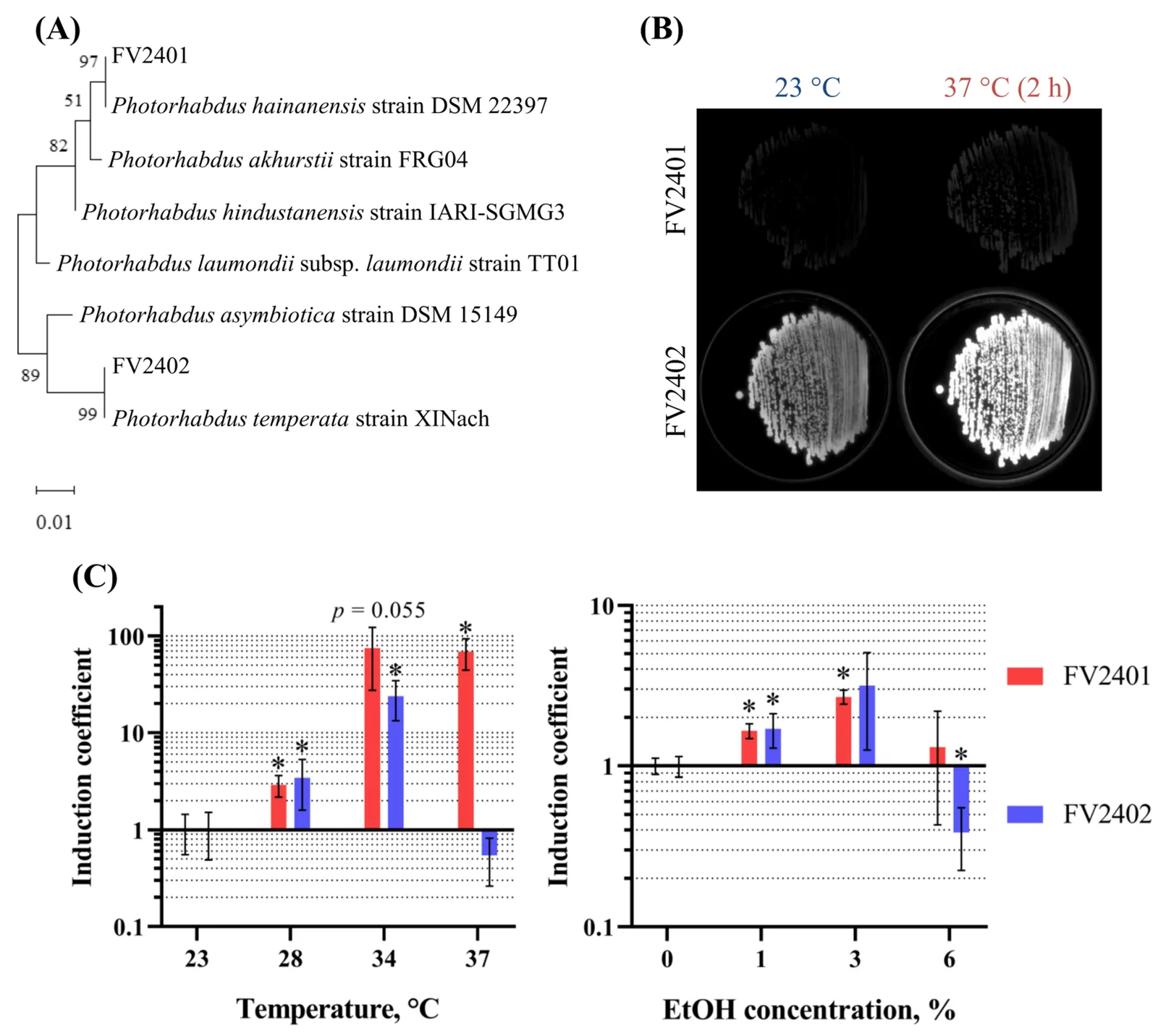
Bioluminescence activation of *Photorhabdus* bacteria. (**A**) Phylogenetic tree based on *16S* rRNA gene sequences for species identification of the isolated strains. FV2401 was assigned to *P. hainanensis*, and FV2402 to *P. temperata*. Bootstrap values are shown at the nodes. (**B**) Basal bioluminescence levels of FV2401 and FV2402 strains incubated on solid LB medium at 23 °C and after exposure of the same plates to 37 °C for 2 h. (**C**) Induction coefficients of *P. hainanensis* FV2401 and *P. temperata* FV2402 after 3 h of incubation at 23, 28, 34, and 37 °C and at 0, 1, 3, and 6% ethanol concentrations. Induction coefficients were calculated as the ratio of luminescence normalized to optical density at a given temperature or ethanol concentration to the corresponding value at 23 °C or 0% ethanol, which were set to 1, respectively. The standard deviation was calculated based on three biological replicates. The statistical significance of the increase in luminescence of samples at temperature (T) or ethanol concentration (ω) relative to 23 °C or 0%, respectively, was assessed using a two-sample *t*-test (H_0_: no increase relative to 23 °C or 0%): *—*p* < 0.05.

To investigate the thermoactivation of the *lux*-operon in *Photorhabdus* species, two novel strains, FV2401 and FV2402, were isolated from tropical and temperate climatic zones, respectively. Phylogenetic analysis based on *16S* rRNA and *infB* gene sequences assigned strain FV2401 to *P. hainanensis* and strain FV2402 to *P. temperata* (Figure 1A and Figure S1). The maximum growth temperatures for the strains did not exceed 37 and 34 °C, respectively (Figure S2). *P. temperata* FV2402 produced stronger bioluminescence than *P. hainanensis* FV2401 on solid LB medium at 23 °C, and upon short-term exposure (2 h) to 37 °C, an increase in bioluminescence was observed in both strains (Figure 1B).

To quantify bioluminescence thermoactivation, induction coefficients (2) were calculated for strains FV2401 and FV2402 cultured in liquid LB to an OD_600_ ≈ 0.1, divided into aliquots, and incubated for 3 h at the indicated temperatures or ethanol concentrations (Figure 1C).

Luminescence in FV2401 was activated at the growth temperatures of 28, 34 and 37 °C, while luminescence in FV2402 was induced at 28 and 34 °C (Figure 1C). At 37 °C, luminescence intensity was higher in FV2401 but not in FV2402, which is consistent with their maximum growth temperatures (Figure S2). At 42 °C, no increase in luminescence was observed for either strain, for the same reason. It should be noted that the FV2401 cell division rate was temperature-dependent with a maximum at 34 °C, while in the case of FV2402 it remained practically unchanged or decreased with an increase in temperature up to 37 °C (Figure S3).

When incubated with ethanol at a concentration of 1%, luminescence induction was approximately the same for both strains (Figure 1C). At 3%, luminescence increased significantly only in FV2401, whereas at 6%, due to ethanol toxicity, the total luminescence level decreased in FV2402.

Thus, luminescence induction in the FV2401 strain, isolated from a tropical region, was temperature-dependent up to 37 °C, whereas in the FV2402 strain, isolated from a temperate region, luminescence was activated at temperatures up to 34 °C. In the presence of ethanol, luminescence was activated at concentrations of 1 and 3% in FV2401 and decreased significantly at 6% in FV2402.

### 3.2. RNA Analysis

**Figure 2:**
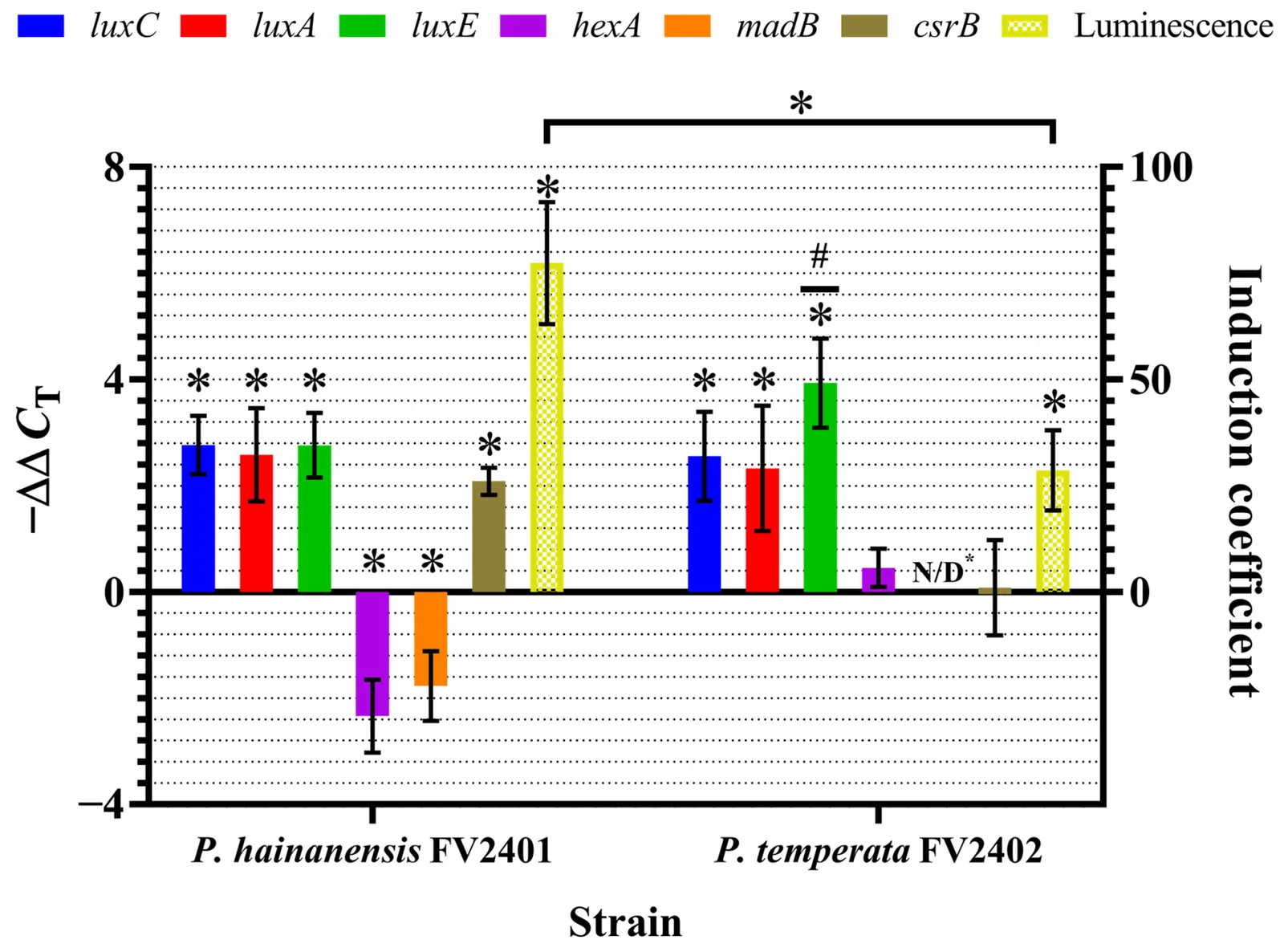
−ΔΔ*C*_T_ values of the *luxC*, *luxA*, *luxE*, *hexA*, *madB*, and *csrB* genes and luminescence induction coefficients in *P. hainanensis* FV2401 and *P. temperata* FV2402 after 3 h of incubation at 37 and 34 °C, respectively. Values are normalized to control samples incubated at 23 °C. The standard deviation was calculated based on three biological replicates. The statistical significance of the changes in mRNA levels was assessed using a one-sample *t*-test (H_0_: there is no induction, ΔΔ*C*_T_ is 0): *—*p* < 0.05. The significance of the difference in luminescence induction between strains was assessed using a two-sample *t*-test (*p* < 0.05). When comparing −ΔΔ*C*_T_ values among the *luxC*, *luxA*, and *luxE* genes, statistical significance was detected only for *luxE*, whose level in *P. temperata* differed from that of the other *lux*-genes (#). N/D*: −ΔΔ*C*_T_ values for the *madB* gene could not be determined in FV2402.

The RNA levels of the *lux*-operon genes *luxC*, *luxA*, and *luxE*, as well as the *hexA*, *madB*, and *csrB* genes, were compared by RT-qPCR in *Photorhabdus* cells exposed for 3 h to heat shock (37 °C for FV2401 and 34 °C for FV2402) relative to cells incubated at 23 °C. The induction coefficients (2) and the −ΔΔ*C*_T_ values (3) of FV2401 and FV2402 are presented in Figure 2.

Luminescence of *P. hainanensis* FV2401 and *P. temperata* FV2402 was induced by increasing temperature, and in the case of FV2401, a more thermostable strain, it was more pronounced (Figure 2). An increase in the mRNA levels of the *lux*-operon genes *luxC*, *luxA* and *luxE* was also observed. −ΔΔ*C*_T_ values for the *luxC*, *luxA,* and *luxE* genes were approximately the same both within and between species, despite the difference in luminescence induction level, except for *luxE* in *P. temperata*.

The mRNA levels of the *hexA* and *madB* genes, which correlate with the *Photorhabdus* cell phenotype, were reduced in FV2401 (Figure 2). In FV2402, however, the *hexA* mRNA level remained virtually unchanged, and the *madB* −ΔΔ*C*_T_ value could not be determined due to the absence of a specific PCR product during RT-qPCR. In turn, the *csrB* mRNA level was significantly increased in FV2401, but not in FV2402 (Figure 2).

As a result, an almost uniform increase in the *luxC*, *luxA*, and *luxE* mRNA levels was observed both within and between FV2401 and FV2402 upon incubation at 37 and 34 °C, respectively, relative to cultures at 23 °C. In contrast, a decrease in *hexA* and *madB* and an increase in *csrB* were observed in FV2401 but not in FV2402.

### 3.3. lux-Operon Promoter Identification and Characterization

The mRNA transcription start sites (TSS) of the *lux*-operon in *P. hainanensis* FV2401 and *P. temperata* FV2402 cells were determined following incubation at 37 and 34 °C, respectively, and at 23 °C. In FV2402, only one TSS was identified at both temperatures, whereas in FV2401, two TSS were detected at 23 °C and three at 37 °C (Figure S4).

**Figure 3:**
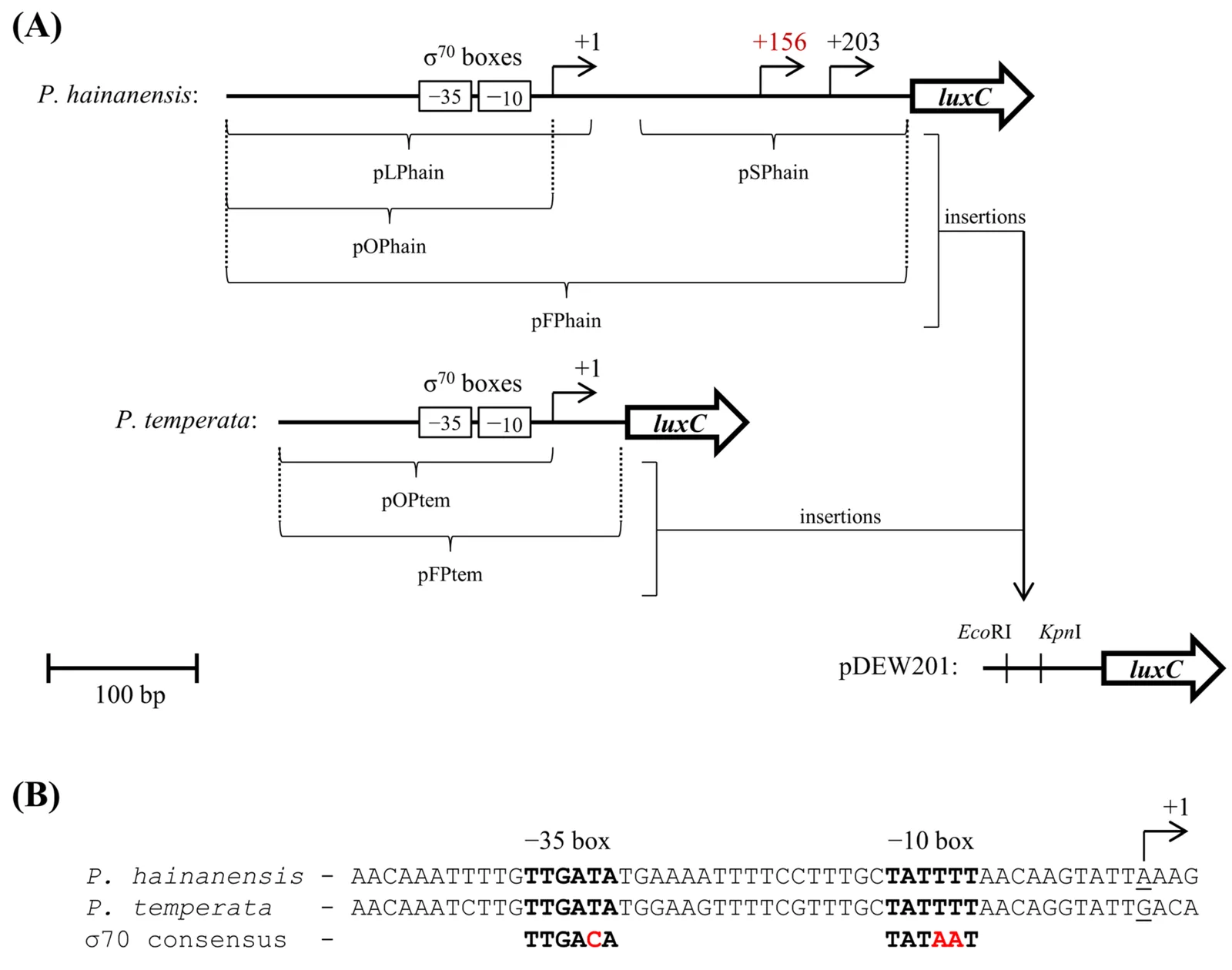
Schematic representation of the *lux*-operon 5′-UTR mRNA of *P. hainanensis* and *P. temperata*. (**A**) Detected transcription start sites (TSS) are indicated by arrows with their nucleotide positions relative to the +1 TSS, upstream of which the −35 and −10 boxes of a σ^70^-dependent promoter were identified. The TSS detected at 37 °C but not at 23 °C is highlighted in red. Below the 5′-UTR mRNA schemes, the inserts cloned into the promoterless plasmid pDEW201 at the *Eco*RI and *Kpn*I sites are shown, with the corresponding names of the resulting constructs indicated. The scale bar is shown at the bottom left. (**B**) Nucleotide sequences upstream of the +1 TSS of *P. hainanensis* and *P. temperata*, aligned with the σ^70^ consensus. The −35 and −10 boxes are in bold, consensus nucleotides that differ from the *Photorhabdus* boxes are highlighted in red, and underlined nucleotides correspond to the +1 TSS.

All TSS were mapped and numbered relative to the +1 nucleotide corresponding to the most distal site upstream of the *luxC* gene, as shown in the scheme presented in Figure 3A. In both species, promoter-like elements were identified only adjacent to the +1 TSS, which is located at distances of 258 nucleotides from the *luxC* start codon in *P. hainanensis* FV2401 and 50 nucleotides in *P. temperata* FV2402 (Figure 3B). These elements correspond to the −35 and −10 boxes, exhibit a high degree of homology between the two species, and are consistent with the organization of promoters recognized by the core σ^70^ subunit of RNA-polymerase [33].

**Figure 4:**
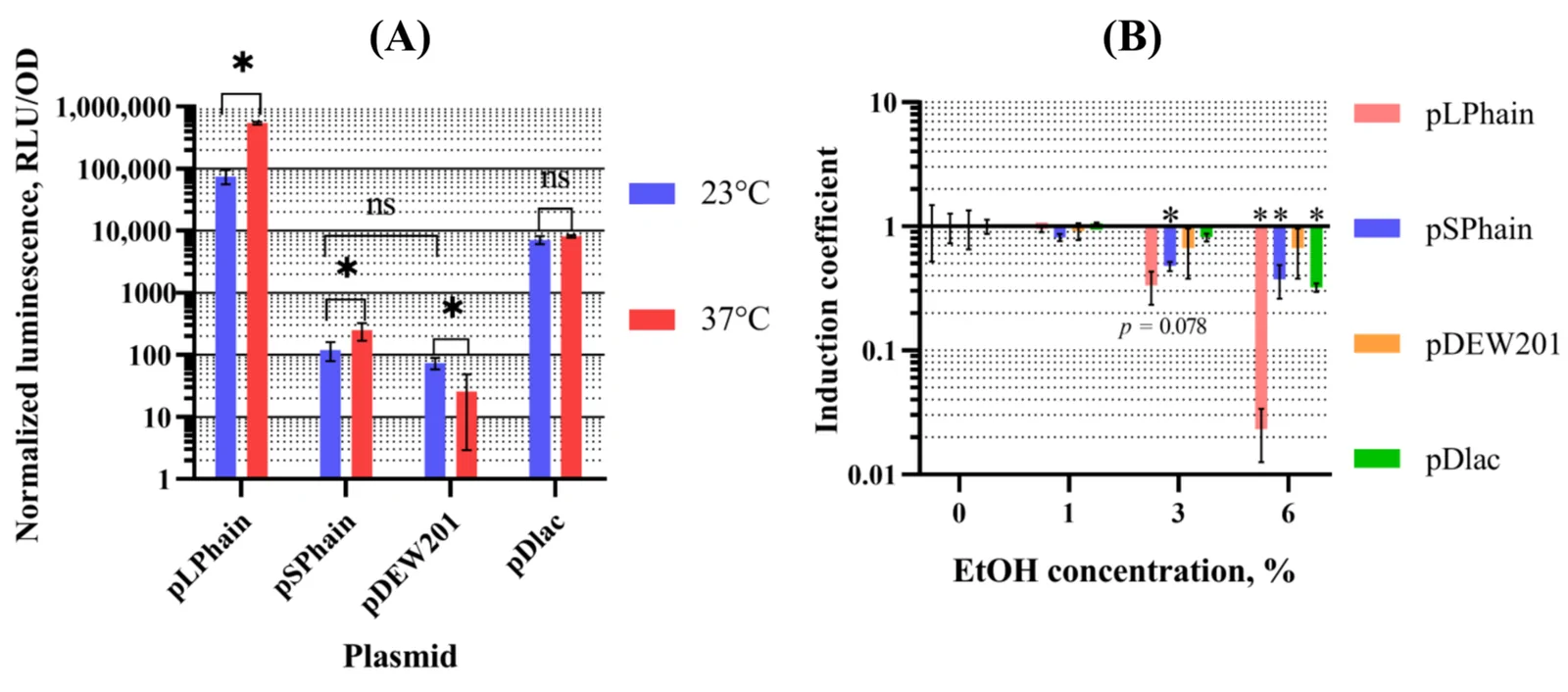
Promoter characterization. (**A**) Normalized to the optical density luminescence of suspensions of *E. coli* MG1655 cells harboring pLPhain, pSPhain, pDEW201, or pDlac plasmids following 3 h of incubation at 23 or 37 °C. The statistical significance of differences between the samples shown was assessed using a two-sample *t*-test: *—*p* < 0.05; ns — not significant. (**B**) Induction coefficients of the same cultures after 3 h of incubation with 0, 1, 3 and 6% ethanol concentrations (0% set to 1). The statistical significance of the increase in luminescence of samples relative to 0% ethanol was assessed using a two-sample *t*-test (H_0_: no increase relative to 0%): *—*p* < 0.05. The standard deviation was calculated based on three biological replicates.

**Figure 5:**
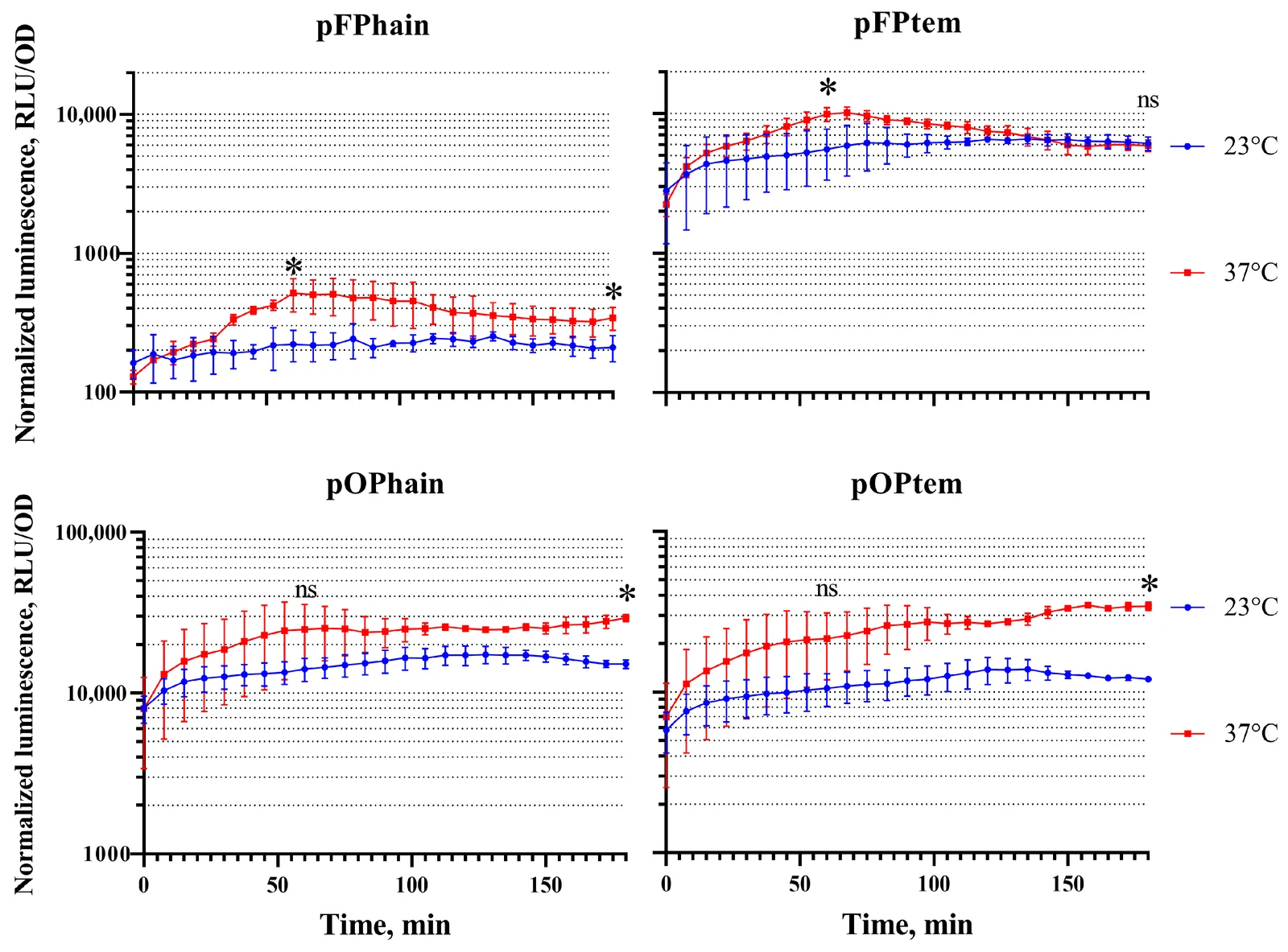
Time-dependent normalized to the optical density luminescence of suspensions of *E. coli* MG1655 harboring pFPhain, pFPtem, pOPhain, and pOPtem plasmids at 23 and 37 °C. Cultures were grown to an initial OD_600_ ≈ 0.1. The standard deviation was calculated based on three biological replicates. Statistical significance was assessed between samples incubated at 23 and 37 °C within the same construct at 60 min and 3 h using a two-sample *t*-test: *—*p* < 0.05; ns — not significant.

To assess the contribution of 5′-untranslated region (5′-UTR) elements to *lux*-operon expression and thermoactivation, different inserts were cloned into the promoterless plasmid pDEW201 and tested for expression in *E. coli* MG1655 cells (Figure 4 and Figure 5). The inserts and the corresponding names of the resulting constructs are given in Figure 3A.

The high degree of homology of the σ^70^ elements between the two species (Figure 3B) prompted us to hypothesize that the promoter adjacent to the +1 TSS is a single, major transcription initiation site in *P. hainanensis* FV2401. To test this hypothesis, luminescence was measured in *E. coli* MG1655 cells harboring pLPhain and pSPhain plasmids, which contain the +1 TSS (identified σ^70^ promoter) and the remaining TSS, respectively (Figure 4). Plasmids pDEW201 and pDlac were used as a negative control.

In the experiment with ethanol (Figure 4B), only a decrease in the luminescence level was observed due to the toxicity of ethanol for *E. coli* cells. Upon temperature induction (Figure 4A), the normalized luminescence (1) of the negative control pDlac remained unchanged. In pDEW201, a promoterless vector, the decrease in normalized luminescence is due to the higher number of cells at 37 °C than at 23 °C, since the cells emit undetectable levels of light at the sensitivity threshold of the luminometer. In contrast, pLPhain exhibited strong luminescence, with an approximately 7-fold induction. pSPhain, by comparison, showed three orders of magnitude lower luminescence and weak induction.. Notably, the normalized luminescence at 23 °C of the promoterless plasmid pDEW201 was practically identical to that of pSPhain.

Overall, only the σ^70^-dependent promoter adjacent to the +1 TSS in FV2401 appears to be involved in *lux*-operon expression and thermoactivation.

To clarify the contribution of σ^70^ promoter and the 5′-UTR mRNA to *lux*-operon thermoactivation, plasmids pFPhain, pFPtem, pOPhain, and pOPtem were constructed. These plasmids contain either inserts carrying both the promoter and the *lux*-operon 5′-UTR mRNA or inserts with only the σ^70^ promoter boxes of the two strains, respectively (Figure 3A). Kinetic curves of luminescence normalized to optical density (1) over time at 23 and 37 °C for *E. coli* MG1655 cell suspensions harboring the pFPhain, pFPtem, pOPhain, and pOPtem plasmids, measured after the cultures reached an OD_600_ ≈ 0.1, are shown in Figure 5.

The normalized luminescence of the pOPhain and pOPtem plasmids, which contain only σ^70^ promoter inserts, increased after 3 h at 37 °C relative to samples at 23 °C (Figure 5). The luminescence intensities of both constructs were of the same order of magnitude (*p* > 0.05), which is not surprising given the sequence homology between the two strains (Figure 3B).

For pFPhain and pFPtem, which also carry the 5’-UTR mRNA, the situation was different. Notably, the luminescence of pFPhain was approximately two orders of magnitude lower than that of pOPhain. However, this was not the case for pFPtem, whose luminescence intensity was almost identical to that of pOPtem.

Another feature was observed for pFPhain and pFPtem: within approximately the first hour at 37 °C, their luminescence increased relative to that at 23 °C, after which it declined. However, after 3 h, luminescence at 37 °C was higher than at 23 °C for pFPhain, whereas for pFPtem the luminescence at 37 and 23 °C coincided. This points to repression of luminescence by elements present in the 5′-UTR mRNA of the *lux*-operon in both species under elevated temperature conditions.

Overall, thermoactivation of the *Photorhabdus lux*-operon in the heterologous *E. coli* system is mediated by a mechanism residing within the σ^70^ promoter region. The construct carrying both the promoter and the 5′-UTR mRNA from the thermotolerant *P. hainanensis* represses background luminescence, whereas the corresponding construct from the thermolabile *P. temperata* does not. In both cases a decrease in luminescence was observed following one hour of thermoactivation.

## 4. Discussion

Temperature induction of the *P. temperata lux*-operon, a representative of a species isolated from a temperate climate, has been shown previously [11]. In the present study, we also carried out experiments on a more thermotolerant representative of the same genus isolated from a tropical zone, *P. hainanensis*. To elucidate the mechanisms of *lux*-operon thermoactivation, we used both species to assess the expression of luminescence-related genes under heat shock, identified the *lux*-operon promoter, and characterized the contributions of the promoter and the 5′-UTR to thermoactivation in *E. coli* cells.

Luminescence of both species, *P. hainanensis* FV2401 and *P. temperata* FV2402, was activated by elevated temperature and by ethanol. Their background luminescence levels differed: in all experiments, FV2402 was brighter than FV2401, as demonstrated on plates. This correlates with data obtained after infection of *Galleria mellonella* larvae [18].

One of the mechanisms involved in *lux*-operon thermoinduction is transcriptional activation, presumably via its promoter [11]. No significant differences in mRNA levels among the *lux*-operon genes were observed in *P. hainanensis* by RT-qPCR. Changes in the transcription of *luxC* and *luxE* genes, which encode components of the fatty acid reductase complex that supplies the substrate to luciferase, were practically the same as those observed for *luxA* gene, which encodes the catalytic subunit of luciferase. This uniform activation of the *lux*-operon genes suggests regulation at the level of the entire gene cluster rather than of individual genes in *Photorhabdus* cells, presumably achieved at the level of transcription initiation rather than termination. In *P. temperata*, the increase in *luxE* mRNA levels was higher than that of *luxC* and *luxA*, suggesting additional regulation.

The higher luminescence induction coefficient observed in *P. hainanensis* compared to *P. temperata* did not correlate with the mRNA levels: the −ΔΔ*C*_T_ values for *lux*-genes in FV2401 were lower than its induction coefficient, whereas *lux*-gene expression should be quadratically related to their mRNA levels, as observed previously in FV2402 and in *E. coli* [11,34]. This indicates that, besides promoter-mediated activation, an additional non-transcriptional mechanism is involved in temperature-dependent bioluminescence activation in *P. hainanensis*, but not in *P. temperata*. Possible primary candidate mechanisms for such activation include the phenotypic state of the cells, as reflected by *hexA* and *madB* expression levels [7,35], and/or *hexA*- and *csrB*-mediated regulation of the *lux*-operon, which operates post-transcriptionally [12,13]. Notably, at elevated temperature, the mRNA levels of *hexA* and *madB* decreased, consistent with their downregulation in brightly luminescent P-form cells and with the repressive function of HexA on the *lux*-operon, while *csrB* RNA increased, which would also be expected to enhance luminescence. Exactly these changes were observed in *P. hainanensis* but not in *P. temperata*.

After RT-qPCR, no PCR product for *madB* was detected in *P. temperata*. Initially, it was assumed that the primers used might not anneal to the target sequences; however, PCR on genomic DNA yielded the expected product, indicating that no methodological error occurred. The most plausible explanation for the absence of *madB* in RT-qPCR is that the cells were initially in the P-form, in which *madB* RNA is not produced due to the inverted orientation of the *mad*-operon promoter [7]. This would account for the presence of the PCR product from DNA but not from RNA. However, the initial phenotypic state in the P-form seems unusual, as P-form cells are expected to arise in stationary phase, typically after two days of incubation at 30 °C [7]. Additionally, the cells were not pigmented, which would also be expected for the P-form [7]. Consequently, only *hexA* expression remained available for monitoring the phenotypic state in *P. temperata*, and its levels did not change with temperature, suggesting a stable phenotype across the incubation conditions. It should also be noted that the regulation of *madB* was studied in *P. luminescens* [7]. Perhaps the regulatory mechanisms of the *mad*-operon differ in *P. temperata*.

The σ^70^-dependent promoter of the *lux*-operon is located at distances of 258 and 50 nucleotides upstream of the *luxC* start codon in *P. hainanensis* and *P. temperata*, respectively. This promoter can also be found in other *Photorhabdus* isolates, with varying distances from the *luxC* start codon (Figure S5). Notably, when comparing the basal luminescence levels of different *Photorhabdus* species [18] with the nucleotide sequences upstream of *luxC*, it appears that species from tropical regions with greater promoter-*luxC* distances exhibit lower basal luminescence than *P. temperata*, where the promoter is located 50 nucleotides upstream of *luxC*. This is also consistent with the observed background luminescence levels of *P. hainanensis* FV2401 and *P. temperata* FV2402 and with the drop in basal luminescence upon cloning of the promoter together with the 5′-UTR mRNA fragment. Moreover, the sequences upstream of the *luxC* are highly variable, except for the regions adjacent to the promoter and in the immediate vicinity of the *luxC*, which parallels the variation in basal luminescence among *Photorhabdus* species [18]. Overall, the inter- and intra-specific differences in basal luminescence are likely mediated by the 5′-UTR of the *lux*-operon, presumably through the formation of different mRNA secondary structures. Basal luminescence levels and 5′-UTR sequences may also be linked to the extent of temperature-dependent bioluminescence activation, as shown for *P. hainanensis* and *P. temperata*. However, given the variability in basal bioluminescence and 5′-UTR, further investigation of a larger number of strains is required, particularly those with intermediate 5′-UTR lengths.

In our previous work, *lux*-operon σ^E^- and σ^32^-independent thermoactivation was considered to occur via melting of mRNA secondary structure [11]. However, the activation of pLPhain, which contains only 42 nucleotides of the *lux*-operon mRNA, raised doubts about this hypothesis. In other bacteria, genes under the control of σ^70^-dependent promoters are known to be activated by temperature [36]. Examples include only chaperone genes, for which a hybrid promoter containing both σ^70^ and σ^32^ boxes [37], or regulation via the CIRCE element, inverted repeats [38,39], have been proposed as regulatory mechanisms. A hybrid promoter can be excluded by the fact that luminescence activation was retained in σ^E^ and σ^32^ mutants [11], and presumably involves an RNA-mediated thermoregulatory mechanism, as it is not induced by ethanol and is retained when only the σ^70^ promoter fragment is inserted into pDEW201. A second type of regulation, mediated by an element located within the mRNA and repressing temperature-dependent activation, also appears to exist, as a decline in luminescence was observed one hour after activation in *E. coli* cells. This regulatory mechanism is probably distinct from CIRCE, since no appropriate inverted repeats were found (Figure S5). The mechanisms regulating *lux*-operon thermoactivation appear to differ from the simplified heat shock described in *E. coli*, although some commonality might be expected given that both *Photorhabdus* and *E. coli* belong to the order *Enterobacterales* and *lux*-operon promoter thermoactivation was observed in *E. coli*. Overall, *lux*-operon thermoinduction resembles heat shock in other bacteria, but is not related to chaperones [36].

It should be borne in mind that the *lux*-operon 5′-UTR mRNA in the pDEW201 plasmid does not retain its native conformation due to the nucleotides between the insertion site and the *luxC* gene, which raises doubts about the relevance of the phenomena observed with pFPhain and pFPtem. Nevertheless, the decrease in luminescence of pFPhain relative to pOPhain (equivalent to pOPtem) mirrors the ratio of basal luminescence of the native strains FV2401 and FV2402, while the decline after one hour of thermoactivation appears to be a general feature. The only point that remains uncertain is the similarity between pFPtem and pOPtem luminescence, since the short *P. temperata* 5′-UTR mRNA insert may be more sensitive to the loss of native sequence context in pDEW201 than the long *P. hainanensis* insert.

In summary, we have shown that thermoactivation of bioluminescence in *P. hainanensis* and *P. temperata* involves both common and strain-specific features. In *P. hainanensis*, the *lux*-operon was transcriptionally activated and regulated as a single gene cluster, whereas in *P. temperata* the increase in *luxE* mRNA levels was more pronounced. In *P. hainanensis*, additional contributions to luminescence activation may arise from changes in the expression levels of *hexA*, *madB*, and *csrB*. A single σ^70^-dependent *lux*-operon promoter was identified in both strains, located at different distances from the *lux*-genes depending on the species. When transferred to the heterologous *E. coli* system, this promoter retained temperature-inducible activity. Cloning of the promoter together with the 5′-UTR from *P. hainanensis* resulted in suppression of background luminescence. In both species, the presence of the 5′-UTR led to a decline in luminescence following an initial increase.

The need to study *Photorhabdus* bioluminescence to better understand its role in ecological and biological functions has been highlighted previously [18]. The present study investigates the molecular mechanisms of bioluminescence activation in symbiotic *Photorhabdus* species from different climatic zones at elevated temperatures, which is of particular interest for understanding bacterial adaptation to environmental stress. The *Photorhabdus lux*-operon promoter, whose activity is regulated by the RNA-polymerase σ^70^ subunit, is activated by heat shock independently of the σ^32^ and σ^E^ subunits sensitive to denatured proteins [11], which justifies its use as a new biosensor sensitive to temperature, but not protein-denaturing agents. This property is useful to determine the specific mechanisms of heat shock condition occurrence under the influence of toxicological substances, as demonstrated in [40]. It is also possible that a *lux*-operon promoter with improved characteristics in response to heat shock might be found among representatives of the genus *Photorhabdus*. Notably, similar mechanisms are likely to apply to human-pathogenic *Photorhabdus* species as well, owing to the presence of the identified promoter (Figure S5) and the previously demonstrated thermoactivation [22].

In conclusion, thermoactivation of bioluminescence in *Photorhabdus* appears to involve one or more regulatory mechanisms, and the extent of activation likely depends on the bacterial environment: it is weak in temperature-sensitive species such as *P. temperata*, stronger in more thermotolerant species such as *P. hainanensis*, and remains uncharacterized in *P. akhurstii*. This activation may be related to a biological function of bioluminescence in the bacteria—if such a function exists—in response to environmental conditions; however, assessing this function requires studies in *Photorhabdus* cells rather than in *E. coli*, and across multiple species due to their variability, ideally using luminescence-deficient strains.

## Data Availability

The data are contained within the article.

## Supplementary Materials

The following supporting information can be downloaded at https://www.mdpi.com/article/10.3390/microorganisms14092076/s1, Figure S1: Phylogenetic tree based on *infB* gene sequences for species identification of the isolated strains. FV2401 was assigned to *P. hainanensis*, and FV2402 to *P. temperata. *Bootstrap values are shown at the nodes; Figure S2: *P. hainanensis* (red segments) and *P. temperata* (green segments) incubated overnight at temperatures of 28, 34, 37, and 42 °C. Photos were taken (**A**) in the light and (**B**) in the dark; Figure S3: Growth coefficients of *P. hainanensis* FV2401 and *P. temperata* FV2402 after 3 h of incubation at 23, 28, 34, and 37 °C. Growth coefficients were calculated as the ratio of the optical density at a given temperature to that at 23 °C, which was set to 1. The standard deviation was calculated based on 3 replicates; Figure S4: Agarose gel with PCR products obtained after 3 rounds of nested PCR using Step-Out RACE method [31]. The left lane of the gel corresponds to the DNA Ladder 100 bp+ (Evrogen); the middle lane contains samples originally synthesized from total RNA isolated from cultures incubated at 23 °C; the right lane from cultures incubated at 37 °C. For FV2401, two PCR fragments were detected at 23 °C and three at 37 °C; for FV2402, a single PCR fragment was detected under both conditions; Figure S5: Multiple alignment of nucleotide sequences upstream of the *luxC* start codon in *Photorhabdus* bacteria. σ^70^ boxes −35 and −10, the +1 nucleotide, and *luxC* start codon are underlined. The distances from the +1 nucleotide to the ATG start codon of *luxC* ranges from 50 to 261 nucleotides; Table S1: *E. coli* and *Photorhabdus* strains used in the study; Table S2: Plasmids used in the study; Table S3: Oligonucleotides used in the study.
