# Supplementary material for: Thermally Induced lux-Operon Promoter of Photorhabdus hainanensis and Photorhabdus temperata
Source: Microorganisms. 2026 Sep 17;14(9):2076. doi: 10.3390/microorganisms14092076 (PMC13609902; doi:10.3390/microorganisms14092076)
Supplement: Supplementary file 1 [file microorganisms-14-02076-s001.zip › microorganisms-4538918-supplementary.pdf]

Supplementary Materials

Thermally Induced *lux*-operon Promoter of *Photorhabdus hainanensis* and *Photorhabdus temperata*

Vadim V. Fomin [1], Valeriia O. Matveeva [1], Kamilla V. Mekhantseva [1], Daniil I. Sakharov [1], Sergei E. Spiridonov [2], Sergey V. Bazhenov [1], Nanette Hope Sumaya [3], Ilya V. Manukhov [1,*]

[1] Moscow Center for Advanced Studies, Moscow, 123592, Russia
[2] A.N. Severtsov Institute of Ecology and Evolution, Moscow, 119071, Russia
[3] Department of Biological Sciences, College of Science and Mathematics, Mindanao State University - Iligan Institute of Technology, Iligan City 9200, Philippines
* Correspondence: manukhovi@mail.ru

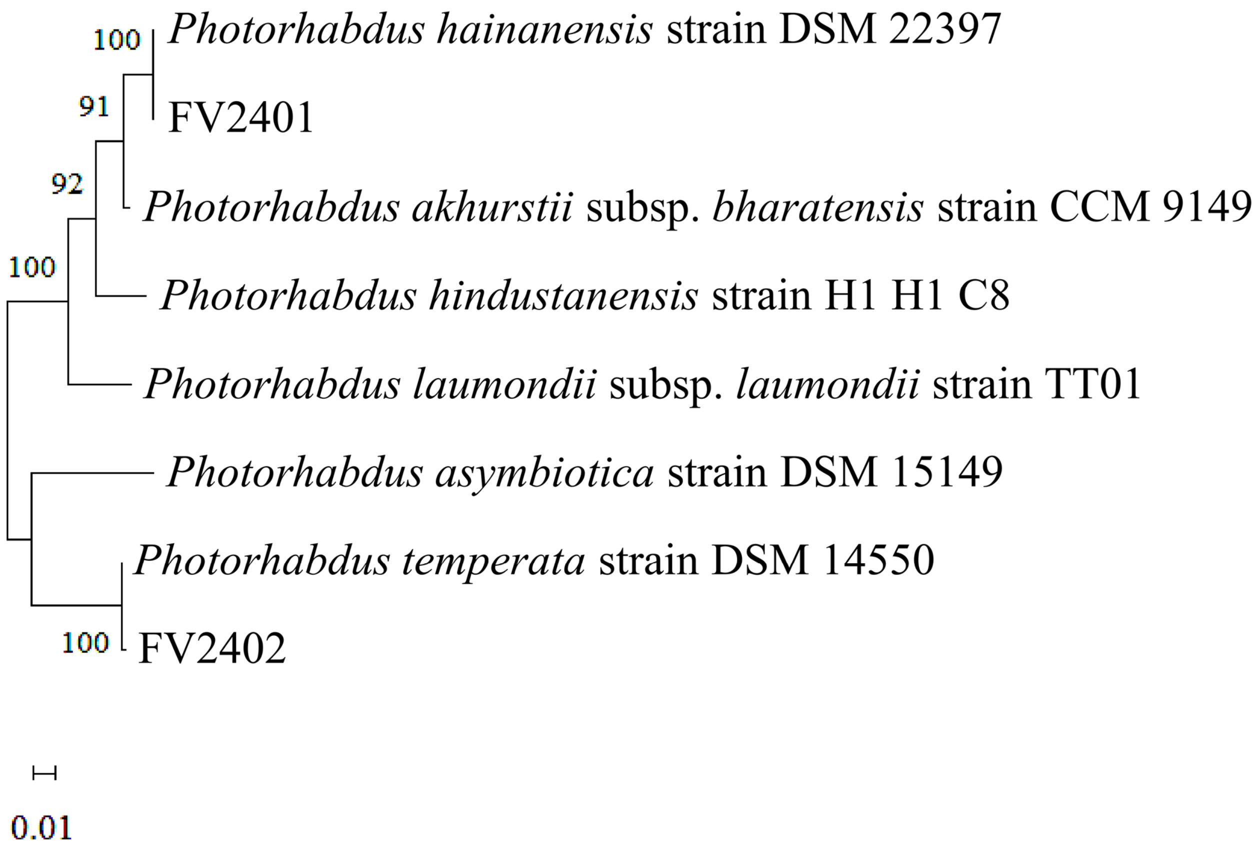

Figure S1. Phylogenetic tree based on *infB* gene sequences for species identification of the isolated strains. FV2401 was assigned to *P. hainanensis*, and FV2402 to *P. temperata*. Bootstrap values are shown at the nodes.

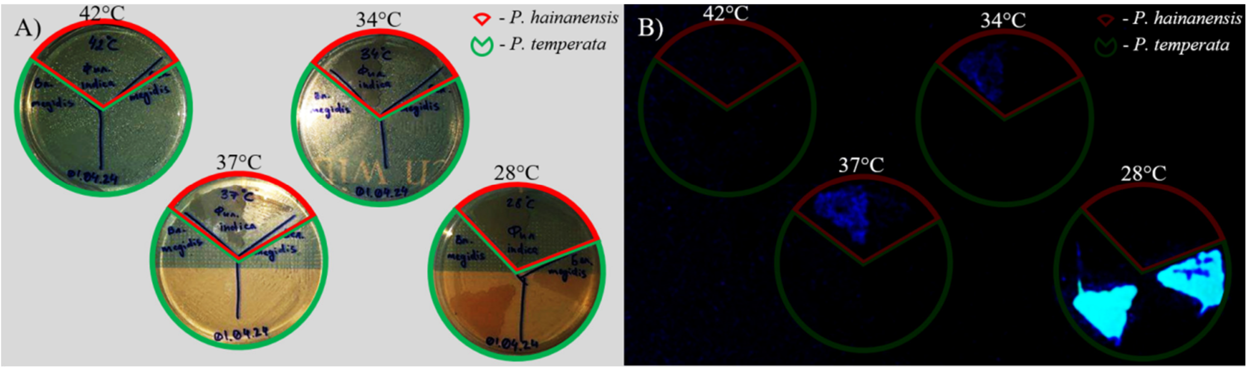

Figure S2. *P. hainanensis* (red segments) and *P. temperata* (green segments) incubated overnight at temperatures of 28, 34, 37, and 42°C. Photos were taken **(A)** in the light and **(B)** in the dark.

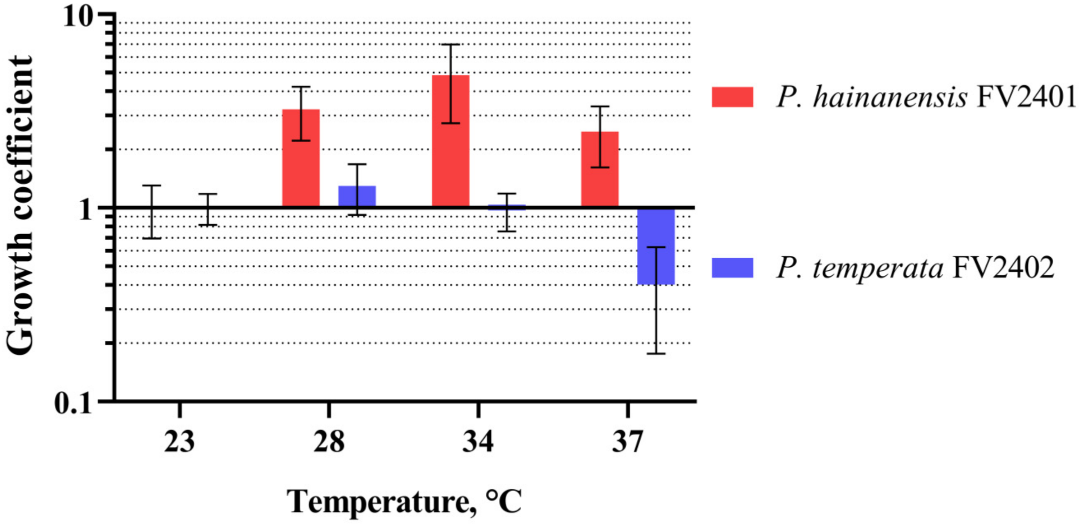

Figure S3. Growth coefficients of *P. hainanensis* FV2401 and *P. temperata* FV2402 after 3 h of incubation at 23, 28, 34, and 37°C. Growth coefficients were calculated as the ratio of the optical density at a given temperature to that at 23 °C, which was set to 1.The standard deviation was calculated based on 3 replicates.

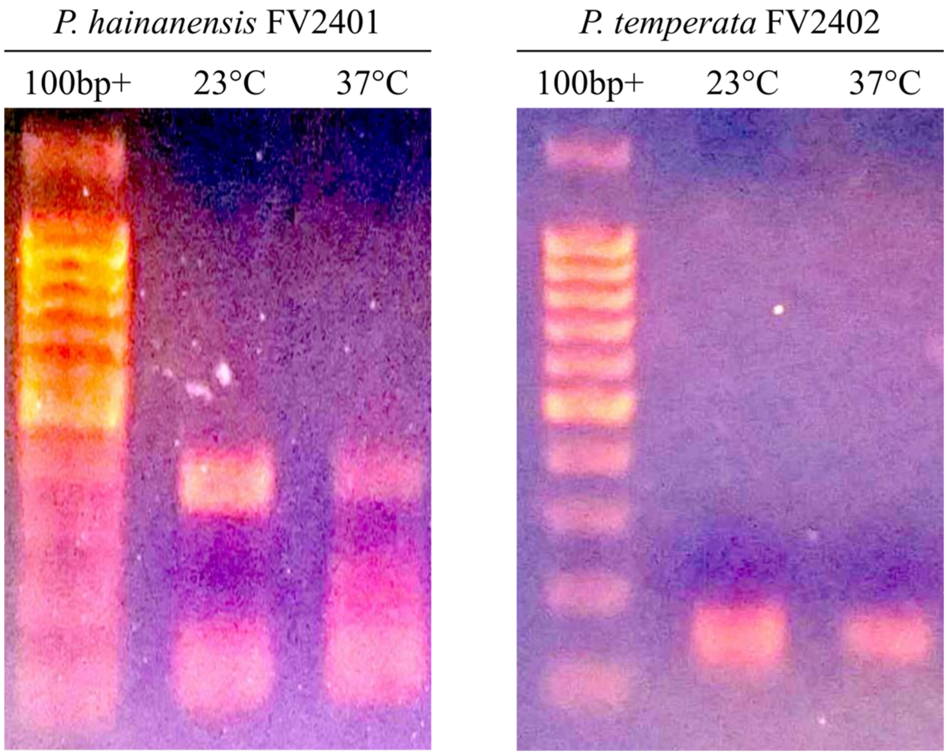

Figure S4. Agarose gel with PCR products obtained after 3 rounds of nested PCR using Step-Out RACE method [1]. The left lane of the gel corresponds to the DNA Ladder 100bp+ (Evrogen); the middle lane contains samples originally synthesized from total RNA isolated from cultures incubated at 23°C; the right lane from cultures incubated at 37°C. For FV2401, two PCR fragments were detected at 23°C and three at 37°C; for FV2402, a single PCR-fragment was detected under both conditions.

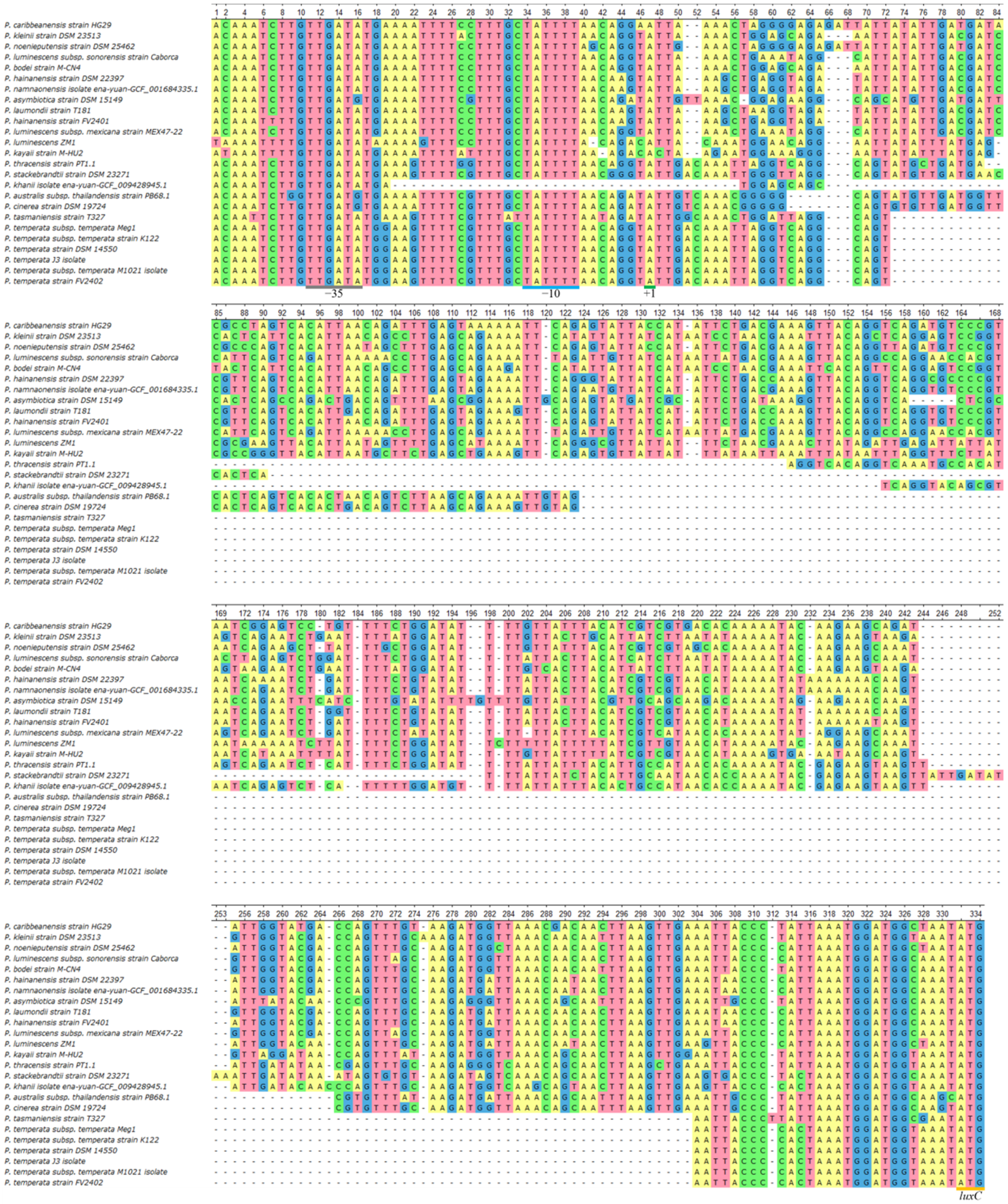

Figure S5. Multiple alignment of nucleotide sequences upstream of the *luxC* start codon in *Photorhabdus* bacteria. σ[70] boxes −35 and −10, the +1 nucleotide, and *luxC* start codon are underlined. The distances from the +1 nucleotide to the ATG start codon of *luxC* ranges from 50 to 261 nucleotides.

Table S1. *E. coli* and *Photorhabdus* strains used in the study.

Strain	Description	Source
E. coli MC1061	F–, *Δ(araA-leu)7697*, *[araD139]*$_{B/r}$, *Δ(codB-lacI)3*, *galK16*, *galE15*(GalS), *λ*$^{-}$, *e14-*, *mcrA0*, *relA1*, *rpsL150*(strR), *spoT1*, *mcrB1*, *hsdR2*	VKPM collection
E. coli MG1655	F–, *λ*$^{–}$, *ilvG–*, *rfb-50*, *rph-1*	VKPM collection
P. hainanensis FV2401	A natural strain isolated from larvae of the small wax moth *Achroia grisella* inoculated with the nematode *Heterorhabditis indica*	Present study
P. temperata FV2402	A natural strain isolated from the greater wax moth *Galleria mellonella* inoculated with nematode *Heterorhabditis megidis*	Present study

Table S2. Plasmids used in the study.

Name	Description	Source
pDEW201	Promoterless plasmid containing the *lux*-operon from *P. luminescens*. Origin of replication pBR322, Ap^r	[2]
pDlac	Plasmid based on pDEW201. The *lac* promoter from *E. coli* MG1655 is inserted, Ap^r	[3]
pLPhain	Plasmid based on pDEW201. The *lux*-operon promoter from *P. hainanensis* FV2401, adjacent to the +1 TSS, is inserted, Ap^r	Present study
pSPhain	Plasmid based on pDEW201. The *lux*-operon promoters from *P. hainanensis* FV2401, adjacent to the +156 and +203 TSS, are inserted, Ap^r	Present study
pFPhain	Plasmid based on pDEW201. The σ^{70} promoter with 5′-UTR mRNA from *P. hainanensis* FV2401 is inserted, Ap^r.	Present study
pFPtem	Plasmid based on pDEW201. The σ^{70} promoter with 5′-UTR mRNA from *P. temperata* FV2402 is inserted, Ap^r.	Present study
pOPhain	Plasmid based on pDEW201. The σ^{70} promoter from *P. hainanensis* FV2401 is inserted, Ap^r.	Present study
pOPtem	Plasmid based on pDEW201. The σ^{70} promoter from *P. temperata* FV2402 is inserted, Ap^r.	Present study

Table S3. Oligonucleotides used in the study.

Name	Nucleotide sequence, 5′ → 3′
16S_Ph_unidir	GGATAACCACTGGAAACGGTGGCTAAT
16S_Ph_unirev	GACTTCATGGAGTCGAGTTGCAGA
infBFor	GCGTGAAGCGGAAGAGCAAG
infBRev	CCACGTGCACGCATTGAAGTAAA
luxC52R	TCGGGAAAGATTTCAACCTGGCCGTTAATA
hainPLF	TGCCAGGAATTGGGGATCGGAAATCTGGTGGTGAAGAGTG
hainPLR	GAATGGCGCGAGCTCGGTACGACTGAACGGATCGTCAATATAA
hainPSF	TGCCAGGAATTGGGGATCGGATCATATTCTGACGAAAGTTACA
hainPSR	GAATGGCGCGAGCTCGGTACTTTGCCATCCATTTAATGGGGTT
hainPOR	GAATGGCGCGAGCTCGGTACTACTTGTTAAAATAGCAAAGGAAAA
pDPtemFor	TGCCAGGAATTGGGGATCGGCTATTGATAATTATGGAGCGTTG
pDPtemRev	GAATGGCGCGAGCTCGGTACTTTACCATCCATTTAGTGGGGTAAT
temPOR	GAATGGCGCGAGCTCGGTACTACCTGTTAAAATAGCAAACGAAAA
Ph_DluxC	GGAAAAATGAAGAATATTCAAGACGCAGG
Ph_RluxC	AAGAGCGCGTTATCGGATGATTAGGG
Ph_DluxA	AAYGGAGTGCTGGTAYGGGTTGATAAA
Ph_RluxA	TTKRTCTGAATCRTCAAAAATGGTCGTGG
Ph_DluxE	AGYAGCGGCACGARTGGTTTAAAAAG
Ph_RluxE	CTTTTCCAGCCKCCCCCCGTTA
DhexA	CTGCGCGCTAACGATGATGCCAG
RhexA	GCTTCATAAGCAATCCGCCACGATAC
DmadB	GTCCTGCGCATCTTAGGCGATACCA
RmadB	GCTGATCACCGCCCATTCCAC
DcsrB	CTGGTTCCATTATGTGTAGCTTGTTTC
RcsrB	GCTCACTCCTTGACAAATTGTCCCT
16SuniD	CGTGCCAGCAGCCGCGGTAATA
16SuniRmidi	GGCCCCCGTCAATTCATTTGAGT
RACE(1)luxC	GCGTCTTGAATATTCTTCATTTTTCCATC
RACE(2)luxC	GGCAGGTAAACACTATTATCACCAAA
RACE(3)luxC	TGAAATTTTTTTACTCATATTTGCCATCC
luxC52f	GCTGGCAATGGTTATTTATTTCGTA

References

1. Matz, M. V.; Alieva, N.O.; Chenchik, A.; Lukyanov, S. Amplification of CDNA Ends Using PCR Suppression Effect and Step-out PCR. *Methods Mol. Biol.* **2003**, *221*, 41–49, doi:10.1385/1-59259-359-3:41.
2. Van Dyk, T.K.; Rosson, R.A. *Photorhabdus luminescens luxCDABE* Promoter Probe Vectors. *Methods Mol. Biol.* **1998**, *102*, 85–95, doi:10.1385/0-89603-520-4:85.
3. Fomin, V.V.; Bazhenov, S.V.; Kononchuk, O.V.; Matveeva, V.O.; Zarubina, A.P.; Spiridonov, S.E.; Manukhov, I.V. *Photorhabdus lux*-Operon Heat Shock-like Regulation. *Heliyon* **2023**, *9*, e14527, doi:10.1016/J.HELIYON.2023.E14527.
